# Open ocean nocturnal insect migration in the Brazilian South Atlantic with comments on flight endurance

**DOI:** 10.7717/peerj.7583

**Published:** 2019-09-18

**Authors:** Ruy J.V. Alves, Luíz A.A. Costa, Alexandre Soares, Nílber G. Silva, Ângelo P. Pinto

**Affiliations:** 1 Departamento de Botânica, Museu Nacional, Universidade Federal do Rio de Janeiro, Rio de Janeiro, Rio de Janeiro, Brazil; 2 Departamento de Entomologia, Museu Nacional, Universidade Federal do Rio de Janeiro, Rio de Janeiro, Rio de Janeiro, Brazil; 3 Laboratório de Sistemática de Insetos Aquáticos (LABSIA), Departament of Zoology, Universidade Federal do Paraná, Curitiba, Paraná, Brazil

**Keywords:** Aeshnidae, Dragonfly, Lepidoptera, Long-distance dispersal, Migration, Nocturnal flight, Pentatomidae

## Abstract

We observed a nocturnal insect swarm aboard the oceanographic ship Cruzeiro do Sul of the Brazilian Navy, while conducting a survey of the Montague guyot (seamount), 389 km distant from the nearest land in the South Atlantic. The insects came from open sea toward the ship from all directions, attracted by the powerful light of the deck. Most insects collided with the hull and fell into the ocean, but we managed to capture and determine 17 (13 Hemiptera of a single species, three Lepidoptera of three species and one Odonata). With one exception, we are certain that none of the specimens caught originated from the ship. The geographic origin, most likely the coast of Brazil, and flight endurance of these insects were inferred using data on wind speed and direction, provided by the crew of the ship, and were reconstructed using Hysplit modeling of air current trajectories.

## Introduction

Insects, with more than one million species, represent two thirds of the total metazoan diversity (Zhang, 2011, 2013). Undoubtedly, their ability to fly may be one of the reasons for their evolutionary success (Grimaldi, 2010). Indeed, winged insects alone correspond to 69% of all animal species. Aside from vertebrates, such as birds and bats, insects are the only extant metazoans that use flapping wings for locomotion. One of their extraordinary capabilities is long-distance flight or migration through the air, both of which have been documented for several groups, including dragonflies, locusts, and butterflies (Holzapfel & Harrell, 1968; Johnson & Bowden, 1973; May, 2013; Chapman, Reynolds & Wilson, 2015). Even though there are many definitions of migration in the literature, there is a general agreement around Dingle’s (2006: 214) concept of migratory behavior as “persistent and straightened-out movement affected by the animal’s own locomotory exertions or by its active embarkation upon a vehicle.” Among the eligible vehicles, the author mentioned the wind.

Insects flying during the night have been more poorly documented than insects flying during the day, in part because night flight is more difficult to observe. Sporadic observations of night flight began as early as the 1930s, with the use of airborne traps (Glick, 1939). Later on, the deployment of an entomological radar, pioneered by Fowler & Lagrone (1968), facilitated such observations. For example, Riley (1975) observed insects flying against the wind, and several more recent studies have found that some insects are capable of active downwind flight (Reynolds et al., 2010a, 2010b, 2016 and references therein). Using entomological radars together with light traps, Feng et al. (2006) documented nocturnal migrations of dragonflies up to 1,000 m. ASL (with the maximum insect densities between 200 and 500 m asl), with air speeds mostly within 10–15 m s^−1^. On a single migration event, they captured 59,342 individuals of *Pantala flavescens* (Fabricius, 1798), and only 96 individuals of *Anax parthenope julius* Brauer, 1865.

There is mounting evidence that many insects use geomagnetic orientation to navigate in the dark and that their migration is active rather than passive (Vácha, 1997, 2015, 2017).

The swarms of Odonata, Hemiptera, and Lepidoptera captured by us on a ship in the open South Atlantic Ocean were attracted to the ships’ light when they were flying several hundred kilometers from the mainland or from any islands. No other ships were detected in the vicinity, which could have been transporting the insects. This lead us to ask, as Reynolds et al. (2010a, 2010b), “*How do insects maintain wind-related orientation … in the dark*?” Several other question came to our minds: how could these insects have flown such long distance in the open ocean. Had they come as stowaways from the Archipelago of Trindade & Martin Vaz (ca 1,200 km from the continent), the closest oceanic island in South Atlantic, where the ship had been a few days earlier? What were those insects doing so far from land? Were they migrating sensu Dingle (2006), or did they accidentally disperse to the open ocean forced by weather extremes? What was the minimal duration of their sustained flights?

We analyze this nocturnal insect swarm by assessing the known natural distributions of the species identified, reconstruction of wind conditions preceding capture, their hypothetical flight paths and duration, and their probable source of origin. This work is based on chance evidence which cannot be replicated easily, but lead us to pose the following main questions: (1) Were those insects truly night migrating? (2) Can the open ocean be a regular migration route for such organisms? (3) What is the land mass source (mainland or island) of these insects?

## Materials and Methods

### Background

Of the Brazilian islands in the South Atlantic, only Trindade (Barth, 1958; Becker, 1998) and Fernando de Noronha (Alvarenga, 1962) have been surveyed by entomologists, in the 1950s and 1960s. Flying insects registered on Trindade are mostly continental species with only 11 species of Lepidoptera, eight true bug species and two species of dragonflies. Among the dragonflies, apparently only *P. flavescens*, a characteristically migrant species and well-known as a successful colonizer (Santos, 1981; Mesquita & Matteo, 1991) is still present in the island of Trindade. Insects endemic to Trindade are *Limonethe beckeri* Costa Lima & Guitton, 1961 (Hymenoptera: Ichneumonidae) and the flightless *Liagonum beckeri* Jeannel, 1961 (Coleoptera: Carabidae).

### Study area and sampling

**Figure 1:**
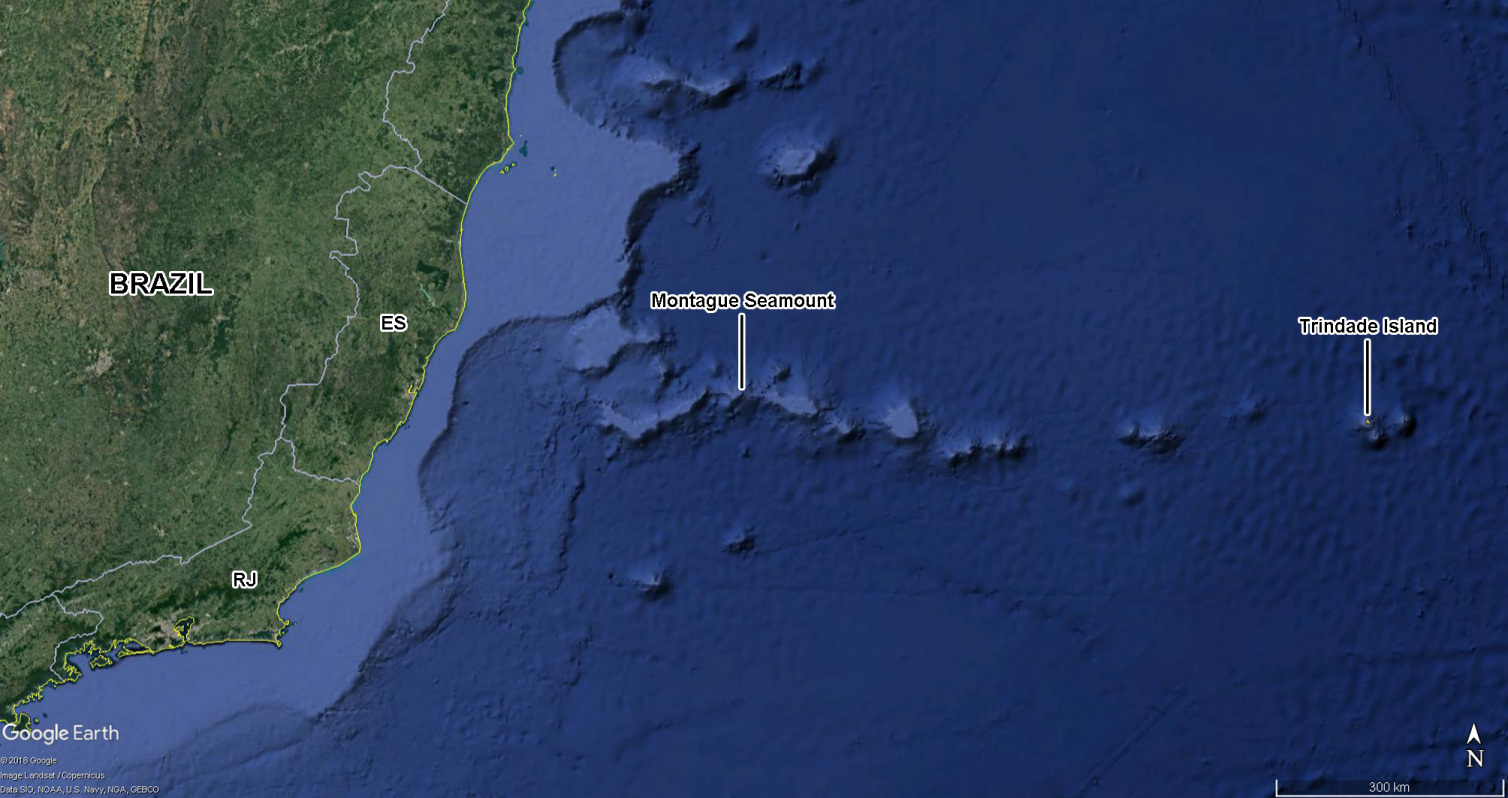
Montague seamount and Trindade Island. Part of South Atlantic Ocean depicting the location of Southern Brazil, Montague seamount (site of anchorage of the hidrooceanographic ship Cruzeiro do Sul of the Brazilian Navy, 20°21′57.60″S, 36°38′46.80″W) and Trindade Island. Abbreviations for the Brazilian States: ES, Espírito Santo; RJ, Rio de Janeiro. Map data: Google, image Landsat/Copernicus, SIO, NOAA, U.S. Navy, NGA, GEBCO.

On May 28, 2014, the ship Cruzeiro do Sul of the Brazilian Navy, on its way from the Archipelago of Trindade & Martin Vaz to the port of Rio de Janeiro, southeastern Brazil, stopped above the Montague seamount (20°21′57.60″S, 36°38′46.80″W), a guyot that is part of the Vitória-Trindade oceanic mountain chain (Fig. 1). This stretch of open ocean is located 389 km from the nearest continental shore, and 764 km from the island of Trindade. The ship halted above the seamount and lowered a measuring apparatus into the water. The white walls of the hull and deck were illuminated by ca. 10,000 Watts of spotlights composed of incandescent, white halogen bulbs (most), and several white fluorescent lights. The maneuver was executed between 22:25 and 23:00 h UTC. Shortly after the lights were turned on, the insects were observed flying straight toward the ship from all directions of open ocean, evidently attracted by the light. Most of them collided against the hull and fell into the ocean, but with the help of crew members and other researchers, two of us (NGS and RJVA) were able to capture and preserve most insects that landed on the deck.

**Figure 2:**
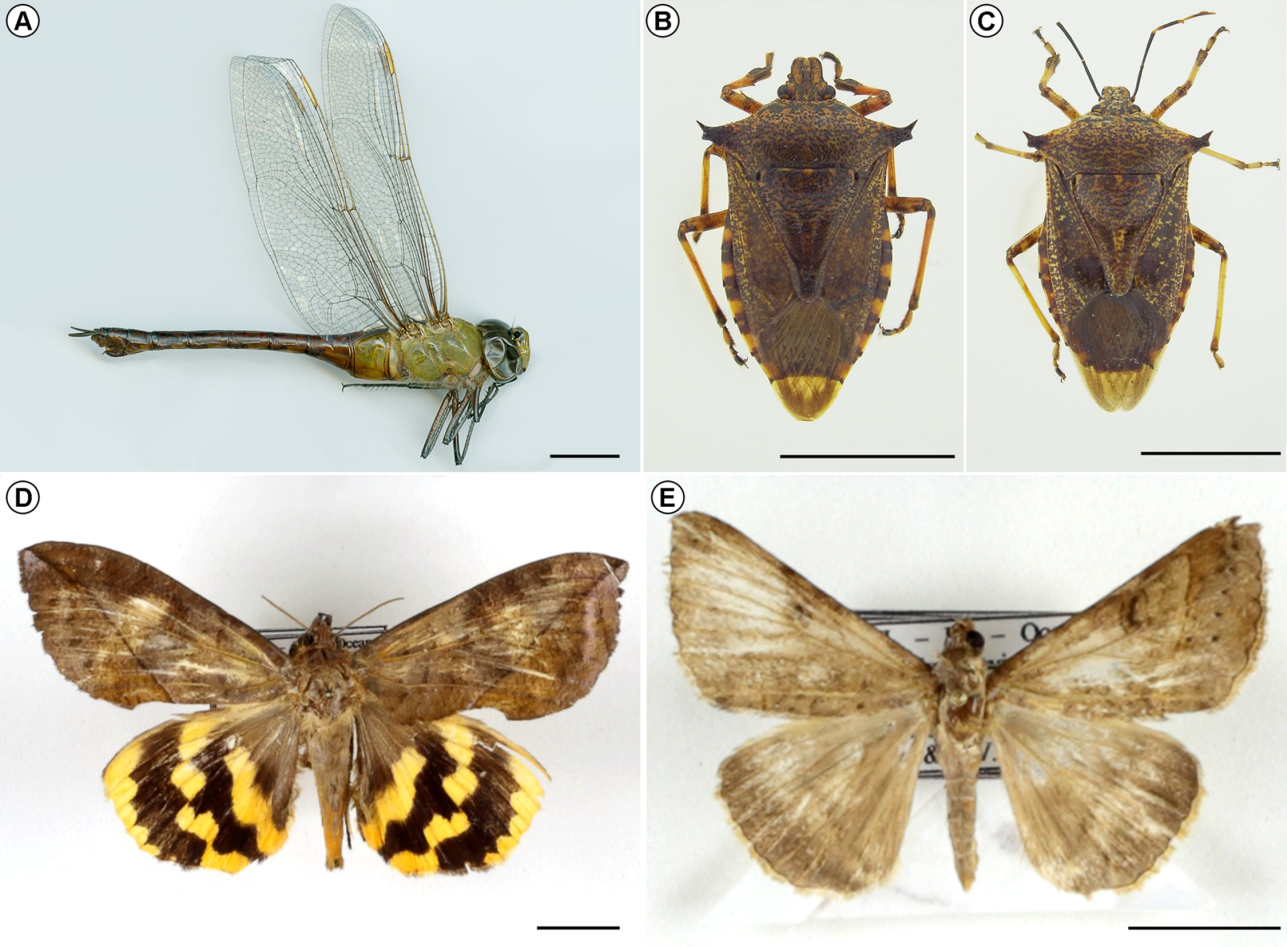
Insects collected at South Atlantic, above the Montague seamount. Habitus of insects collected at South Atlantic, above the Montague seamount, during a nocturnal swarm flight. (A) Female of *Anax amazili* (Burmeister, 1839) in lateral view, (B) and (C). Male (B) and female (C) of *Alcaeorrhynchus grandis* (Dallas, 1851) in dorsal view, (D) male of *Eudocima procus* (Cramer, 1777), (E) *Mocis repanda* (Fabricius, 1794). Scale bars = 10 mm. Photo credits: Ângelo P. Pinto (A–C) and Ruy J.V. Alves (D) and (E).

The Hemiptera and Odonata were preserved mounted, or put in envelopes and deposited in the Department of Entomology of the Museu Nacional (MNRJ), Universidade Federal do Rio de Janeiro, Rio de Janeiro, Brazil. However, on September 2, 2018, the main building of the MNRJ burned down and most of the collections housed there, roughly estimated at 20 million research items, were lost (see Zamudio et al., 2018). This includes the Hemiptera and Odonata reported here (Figs. 2A–2C). Pending determination of one species, the Lepidoptera were kept in paper envelopes placed in the Botany department, thus surviving the fire tragedy. The captured insects were determined by the co-authors LAAC (Hemiptera), AS (Lepidoptera) and APP (Odonata). All material was collected following the Brazilian biodiversity policies, under Instituto Chico Mendes de Conservação da Biodiversidade—ICMBIO/SISBIO, (license numbers 25034-6 and 41194-4).

### Analysis

The crew provided us with records of the wind direction and intensity for a period of 21 h prior to the capture event. This was essential to estimate the trajectory which a hovering insect could have performed during that time. The capture occurred on the night immediately preceding the new moon, hence the night was very dark, and our ship was the only bright light source in a vast region of open ocean. The air temperature measured on deck was 25.4 °C.

We first reconstructed changes in wind speed and direction using the ship’s log data and calculated the trajectory which an idle floating object would have performed spanning 21 h prior to capture. We calculated the distance between the closest coastal point in Brazil and our GPS (datum WGS84) coordinates at the capture site.

Subsequently, we reconstructed 24 backward trajectories preceding the capture by 34 and 48 h and forward trajectories 180 h toward the African coast using Hybrid Single Particle Lagrangian Integrated Trajectory Model (Hysplit, Stein et al., 2015) into a web-based system (Rolph, Stein & Stunder, 2017), not leading into account heading deviations by active flight of the insects. Hysplit settings used: *Compute archive trajectories; Type of Trajectory = Ensemble*; *Meteorology file = Reanalysis (global, 1948–present)*; *Backward/Forward trajectory direction; runtimes (34, 48 or, 180 h); altitudinal range 10–3,000 m agl*.

## Results

### Flight endurance

**Figure 3:**
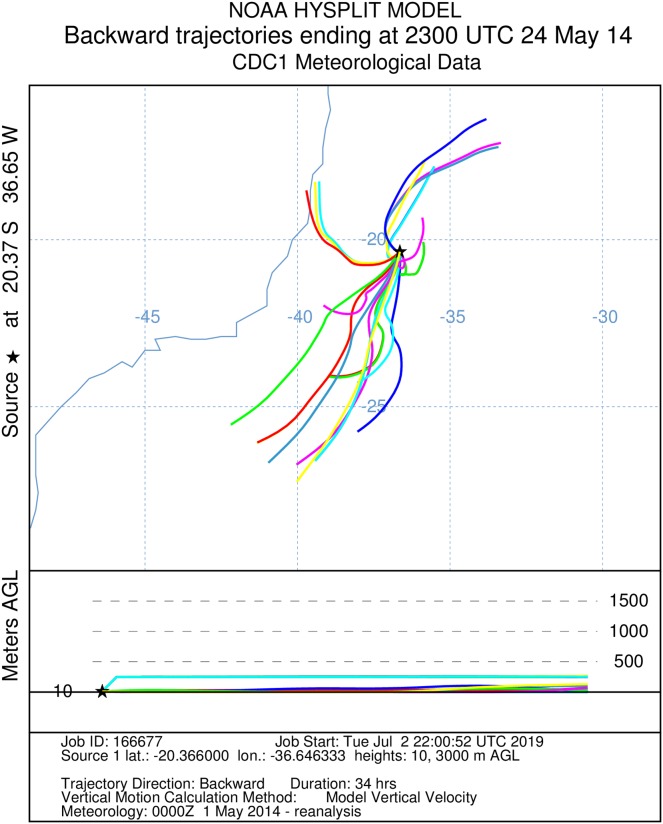
A total of 34 h backward Hysplit reconstruction of wind vectors. Potential origin of captured insects within 34 h. Backward Hysplit reconstruction of wind vectors leading toward to the capture point. Note: single vector originated on the coast at the border of the states of Bahia and Espírito Santo.

**Figure 4:**
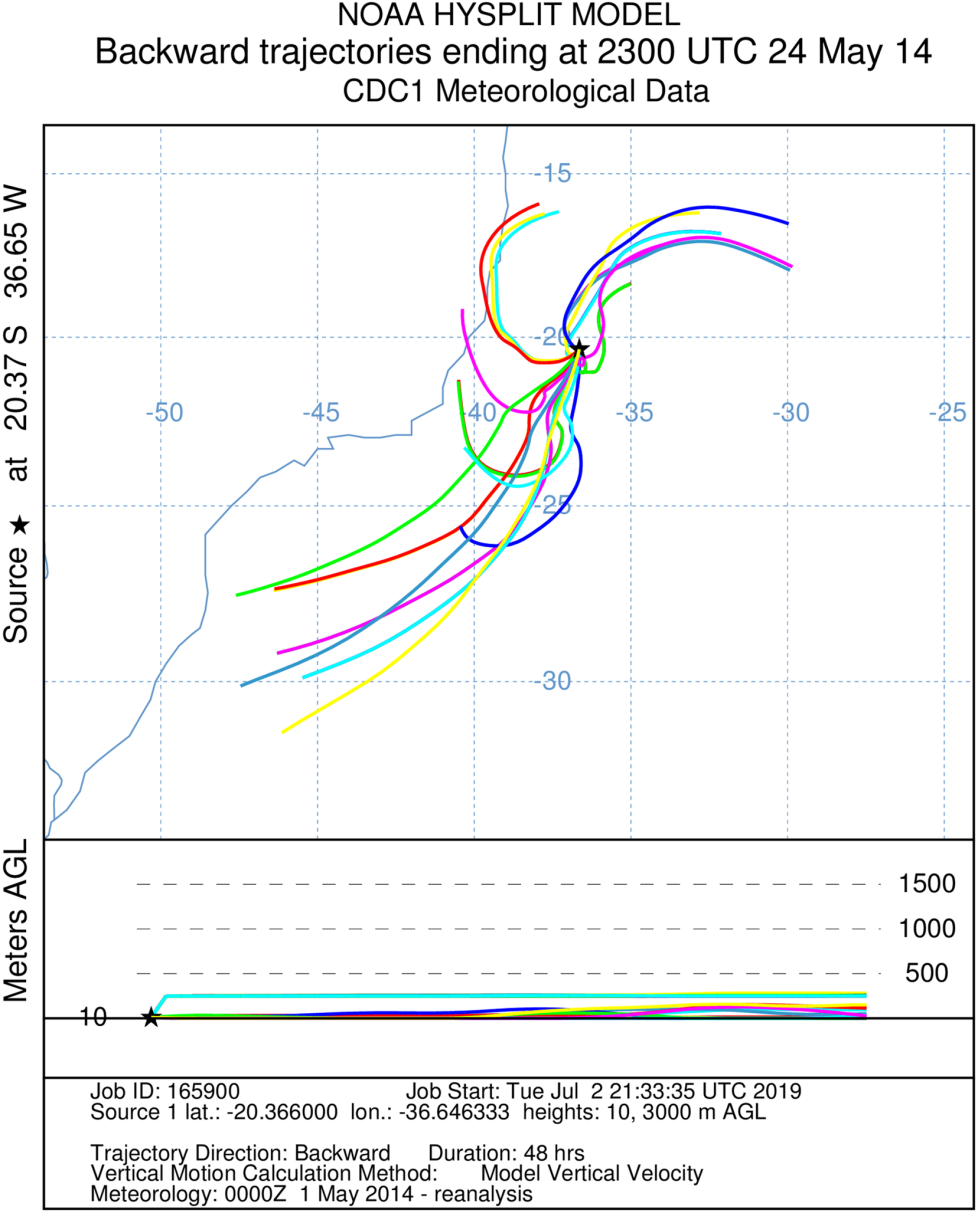
A total of 48 h backward Hysplit reconstruction of wind vectors. Potential origin of captured insects within 48 h. Backward Hysplit reconstruction of wind vectors leading toward to the capture point. Note: three vectors originated on the coast of Espírito Santo and Bahia.

**Figure 5:**
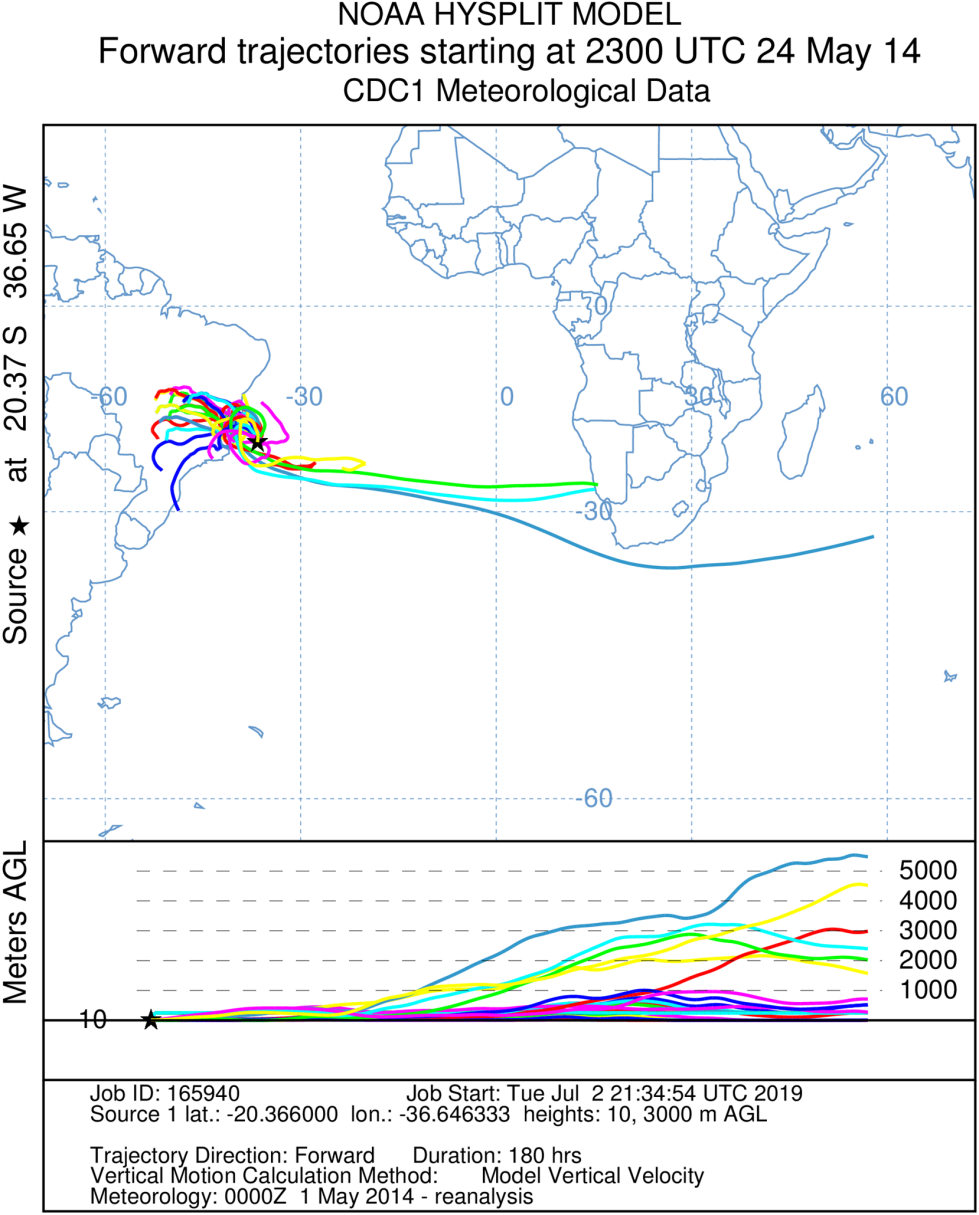
A total of 180 h forward Hysplit reconstruction of wind vectors. Potential dispersal from South America to Africa. Forward 180 h Hysplit reconstruction of wind vectors leading from to the capture point. Note: two vectors arriving in Namibia on the African coast.

It was not possible to calculate the active flight speeds of the sampled insect species. According to the reconstructions of wind trajectory, the minimum “flight time” from the continent (considering that insects were carried passively) was 34 h when a single backward trajectory started on the coast between the Brazilian states of Espírito Santo and Bahia, 380 km from the point of capture (Fig. 3). Three additional potential points of origin on the coast were reconstructed for a 48 h-flight duration (Fig. 4). If the migration of the insects were to continue toward Africa from the point of capture, assisted by the prevailing winds, another 180 h would be needed, and the insects would need to gradually ascend to higher altitudes following the air currents (Fig. 5).

### Insects over the open ocean

There are no species endemic to Trindade island among the insects we captured. Hemiptera were the most numerous, with 13 individuals of only one species, several of which emitted a strong odor resembling green apples, an odor still present in 2017, after 3 years of preservation (subsequently lost in the fire). Three species of Lepidoptera are all moths. A single very large dragonfly completes the list.

**Odonata: Anisoptera: Aeshnidae**

**1. *Anax amazili* (Burmeister, 1839)**
(Fig. 2A)

**Material examined.** One female (MNRJ ODO-0012, subsequently lost in the fire).

**Remarks.** Species of *Anax* are known as migratory. They spend many weeks in migration routes undertaking distinct behaviors such as foraging (cf., May, 2013). *Anax amazili* is a New World species widespread in the entire American continent occurring from the USA (Needham, Westfall & May, 2000) through central America and the Caribbean Islands (Calvert, 1905) to southern Argentina (von Ellenrieder & Muzón, 2008). It also occurs as a resident species in the Pacific archipelago of Galápagos, about of 1,000 km from the coast (Calvert, 1905; Peck, 1992). The site of capture was about 380 km from the Brazilian coast and almost 800 km west of the closest archipelago, Trindade & Martin Vaz, from which only two different Odonata were recorded to date (MNRJ): *P. flavescens* and *Rhionaeschna* sp. (see Santos, 1981). The pantropical Libellulidae glider *P. flavescens* in the best-known migrant dragonfly species (Samways & Osborn, 1998). The hypothesis that this species forms a global panmictic population has received recent support (Troast et al., 2016). However, it is not known if there is a regular migration route among these land masses.

**Hemiptera: Heteroptera: Pentatomidae**

**2. *Alcaeorrhynchus grandis* (Dallas, 1851)**
(Figs. 2B and 2C)

**Material examined.** 12 specimens, four males, eight females, including a pair *in copula*. Many more were seen drowning alongside the deck.

**Remarks.** Recorded from Brazil, Colombia, Mexico, and the southern United States (Ribeiro et al., 2010); Argentina, Bolivia, Cuba, Ecuador, Paraguay and the Galapagos (Dellapé, Martínez & Coscarón, 2003). In Brazil there are collections from Bahia: (sine loco); Goiás: (Chapada dos Veadeiros); Minas Gerais: (Belo Horizonte—sucking a caterpillar); Mato Grosso: (sine loco); Espírito Santo (20°40′S 40°30′W); Rio de Janeiro: Angra dos Reis; Petrópolis; Rio de Janeiro (Copacabana; Quinta da Boa Vista); Serra dos Órgãos; Paraná: (sine loco); Santa Catarina: (sine loco); São Paulo: (sine loco) (MNRJ collection, subsequently lost in the fire); Pará (Ribeiro et al., 2010); Rio Grande do Sul (Dellapé, Martínez & Coscarón, 2003; Magistrali et al., 2014).

**Lepidoptera: Erebidae**

**3. *Eudocima procus* (Cramer, 1777)**
(Fig. 2D)

**Material examined.** One male/female

**Remarks.** Originally described from Surinam (Zaspel & Branham, 2008), this species has a widespread distribution in the Neotropics (Zilli & Hogenes, 2002); in Brazil, it occurs at least in the states of Santa Catarina and Rio Grande do Sul (material previously deposited in MNRJ).

**4. *Mocis repanda* (Fabricius, 1794)**
(Fig. 2E)

**Material examined.** One specimen.

**Remarks.** Widespread in South America: Argentina, Bolivia, Brazil, El Salvador, Colombia, Guyana, Mexico, Nicaragua, Surinam, Venezuela, West Indies (Muthaiyan, 2009). Also found in Central America and the Caribbean, including Cuba, the Dominican Republic, Guadeloupe, Martinique, Guatemala, Jamaica, Puerto Rico, and Saint Thomas; strays can be found in the United States, up to southern Texas as well as subtropical Africa south of the Sahara, including the islands of the Indian Ocean (De Prins & De Prins, 2017). The species was registered from Trindade island up to the late 1950s (Barth, 1958).

**5. Undetermined species (damaged specimen)**

**Material examined.** One specimen.

## Discussion

### Night migration and ocean as route

One of the most interesting questions about insects flying over open ocean on a moonless night so far from any land is whether they (and if so, which ones) were flying toward a direction they chose or changed directions in response to changing environmental factors, for example, geomagnetic cues (Vácha, 1997, 2015); the time-compensated sun-compass (Reppert, Gegear & Merlin, 2010; Guerra et al., 2012) or the wind cues described by Reynolds et al. (2010a, 2010b, 2016). How, if at all, did our sampled insects orient themselves before they were attracted to and actively flew toward the ship’s lights at night? Chapman, Reynolds & Wilson (2015) divided night-flying orientation by insects into two main categories random and many distinct versions of wind-related, but did not mention moon or star light as sources of orientation for insects flying at night. The fact that hundreds of insects actively pursued the ship in the dark of a moonless night suggests that light is a stronger cue to their orientation than the wind characteristics, as proposed by Reynolds et al. (2010a, 2010b).

Geijskes (1968) considered the dragonfly *Anax amazili* as having predominantly crepuscular behavior and sometimes being attracted by light at night. However, this species presents a more complex behavior than previously expected. In the state of Rio Grande do Sul Brazil (30° south latitude), it was collected in light traps and was observed foraging and ovipositing in small open swamp areas at noon (AP Pinto, 2004, personal observation). Our finding shows that this species is capable of sustained night flight. However, if this specimen was truly migrating, where was it heading? After all, the known distribution includes neither Africa nor the islands in the South Atlantic. However, whether the individuals of *Anax amazili* started to migrate to move away from unsuitable environmental conditions or to colonize a new place in the migration arena (sensu Dingle & Drake, 2007) cannot be ascertained based on the available data.

Glick (1939), in his interesting study collecting insects from airplanes, did not register any Odonata flying at night. However, crepuscular or even nocturnal flight are not uncommon to in these insects (Feng et al., 2006; Corbet & Brooks, 2008; May, 2013). Some species of the genus *Anax* are well-known as migrants (May, 2013), but little is known about their flight activity periods. Wikelski et al. (2006) attached miniaturized radio transmitters to 14 individuals of the related *Anax junius* (Drury, 1770 [1773]), and followed their migration for 12 days, using receiver–equipped airplanes and ground teams. They found an average net advance of 11.9 ± 2.8 km/day, exclusively during the day time. Hence our record of *Anax amazili* migrating during the night might well be the first. Extrapolating the flight speed of this species to *Anax amazili*, considering the nearest reconstructed continental point of origin, sustained flight preceding capture would have lasted 33 days, including nights. However, aspects of the migration of *Anax junius* such as a stopover (stationary period for resting or foraging) of about 3 days and the fact that migration does not take place when the wind speed is higher than 25  km/ h (Wikelski et al., 2006) argues against this extrapolation. Therefore, how *Anax amazili* obtained energy during the flight, if it was from storage sources or even feeding on the wind-borne small preys, is unknown. The measured distance in the long-range migration was up to 140 km per day for *Anax junius*, carrying the weight of the transmitters (see discussion in May, 2013), in addition it was recorded flying about of 16 km/h under cool conditions (<22 °C) during migration (May, 1995), speed that allows cover about 400 km in a single day. It is therefore very likely that *Anax amazili* spending just 24 h or few days, since assisted by wind, to cover almost 400 km in open ocean.

The stink bug, *Alcaeorrhynchus grandis*, was considered an introduced or invasive species in the oceanic archipelago of Galápagos, Ecuador, where it was first recorded in 1937 and was argued to have arrived by passive transport on plants to Floreana Island (Peck et al., 1998), distant just over 1,000 km from continental South America. Our observation of *Alcaeorrhynchus grandis* in the open ocean provides support for the hypothesis that this insect could have migrated actively to the Galápagos. However, to support this hypothesis, these events should be dated by means of population and migration studies and modeling of air currents. In addition, dozens of recently dead individuals of *Alcaeorrhynchus grandis* were collected on the shoreline of a beach in the state of Santa Catarina, southern Brazil, in January of 2019 (AP Pinto, personal observation). All this suggests that this species is able to migrate over large obstacles such as ocean. Flying over obstacles such as large bodies of water is uncommon among migratory species (Anderson, 2009).

Insects flying at night often align their flight paths with the wind (Riley, 1975; Reynolds et al., 2010a, 2010b, 2016). Whether the insect’s bodies are heading upwind or downwind during such aligned night flights probably depends on the wind speed and has been subject to some debate. Several studies, contrary to Riley (1975)’s observations, indicate that the insects increase their speed by actively traveling downwind (Reynolds et al., 2010a, 2010b, 2016). In the Northern Hemisphere, Reynolds et al. (2016) found that insects tend to fly slightly to the right in relation to the wind axis and align themselves with the flow direction with greater accuracy with increasing flight altitude.

The three Lepidoptera we captured were noctuid moths. A close relative known to fly long distances is the monarch butterfly. Monarchs can fly in still air at average speeds of 37.5 km/h and probably up to 50 km/h. Even though they usually fly close to the ground, there are records of them flying at altitudes of ca. 3,500 m and flying for more than 600 km, non-stop, for about 16 h over the water (Poirier, 1995). Even though Monarchs are considered day butterflies, a part of that 16 h flight would obviously happen in the dark. On the other hand, the captured moths are active at night. If Monarch butterflies can fly ca. 400 km from the continent to a site of capture in just under 11 h of sustained flight, it is quite possible that other Lepidoptera can also reach the site in comparable speeds, and that migration across the entire Atlantic from South America to Africa could be achieved in 3.6 days in still weather. If winds are factored into the equation. The migration could potentially be considerably faster due to predominant winds, or slower during the rarer reversals when winds blow from Africa to South America.

Even though some insects have been observed resting on the surface of a calm ocean and resume flight after that (Walker, 1931 and references therein), the ocean conditions during our capture were three on the Beaufort scale—hardly considered calm.

### Land mass source (mainland or island)

Of the species captured by us, the moths *M. repanda* (a widespread species considered a pest of various grasses including corn) and *E. procus* were also collected in Santa Catarina and Rio Grande do Sul (SpeciesLink (CRIA), 2017). *Mocis repanda* was registered as the most abundant moth in the Trindade island in 1957 (Barth, 1958), and probably persisted there in the 1960s (Becker, 1998). Hence there is a remote chance that the individuals belonging to *M. repanda* hitchhiked from that island (either flying or stowed in cargo), flew away prior to being captured, and then returned toward the suddenly illuminated ship in the open ocean. Even based on the observed event favoring the hypothesis of active flight hundreds of kilometers from the closer land mass, at least for these moths we cannot discard that they were already on the ship and started to fly after ships’ lights were turned on.

The two other captured insect species (the stink bug and the dragonfly) occur in Brazil, some also extending to countries north and south, but apparently none to Africa. Combining the ranges of distribution with reconstructions of wind speed and direction during the days preceding capture, it is hence reasonable to suppose that all insects captured began their flight in Brazil, somewhere on the coast between Bahia and Espírito Santo.

## CONCLUSIONS

Migration occurs across a series of organizational levels and includes extrinsic (environmental conditions) and intrinsic (behavioral and genetic) elements, even though it is recognized as a single phenomenon (Dingle & Drake, 2007). Migration in insects is an intriguing issue and involves a complex suite of processes and should be investigated using distinct tools. From this unique event reported here, the number of species of insects flying at night and possibly migrating long distances over the open ocean is expanded. Many species avoid crossing large water bodies (Anderson, 2009). In conclusion, our data show that at least the species of stink bug (Pentatomidae) and the dragonfly (Odonata) are capable of flying at night and migrating over obstacles such as large water bodies. Key aspects that need to be further addressed include: (1) whether these insects are mandatory night-flying migrants and eventually perform migration by day. (2) What are there are physiological and population mechanisms promoting the migration syndrome? (3) In case of active migration, could the insects keep a direct course both during the day and at night? (4) How long could the insects fly nonstop before exhaustion?

## Additional Information and Declarations

### Field Study Permissions

The following information was supplied relating to field study approvals (i.e., approving body and any reference numbers): All material was collected following the Brazilian biodiversity policies, under Instituto Chico Mendes de Conservação da Biodiversidade—ICMBIO/SISBIO (license numbers 25034-6 and 41194-4).

### Data Availability

The following information was supplied regarding data availability: All data are available in the article including the parameters used in the Hysplit model of wind vectors.
